# The serotonin receptor 5-HT2A modulates lifespan and protein feeding in *Drosophila melanogaster*

**DOI:** 10.3389/fragi.2022.1068455

**Published:** 2022-12-01

**Authors:** Allyson S. Munneke, Tuhin S. Chakraborty, Saige S. Porter, Christi M. Gendron, Scott D. Pletcher

**Affiliations:** ^1^ Program in Cellular and Molecular Biology, University of Michigan, Ann Arbor, MI, United States; ^2^ Department of Molecular and Integrative Physiology, University of Michigan, Ann Arbor, MI, United States; ^3^ Gertiatrics Center, University of Michigan, Ann Arbor, MI, United States

**Keywords:** *Drosophila melanogaster*, serotonin, nutrients, protein, genetics, lifespan

## Abstract

The conserved neurotransmitter serotonin has been shown to be an important modulator of lifespan in specific nutritional contexts; however, it remained unclear how serotonin signaling influences lifespan under normal conditions. Here, we show that serotonin signaling through the 5-HT2A receptor influences lifespan, behavior, and physiology in *Drosophila*. Loss of the 5-HT2A receptor extends lifespan and induces a resistance to changes in dietary protein that are normally detrimental to lifespan. 5-HT2A^*−/−*^ null mutant flies also display decreased protein feeding and protein content in the body. Therefore, serotonin signaling through receptor 5-HT2A is likely recruited to promote motivation for protein intake, and chronic reduction of protein-drive through loss of 5-HT2A signaling leads to a lower protein set-point adaptation, which influences physiology, decreases feeding, and increases lifespan. Our findings reveal insights into the mechanisms by which organisms physiologically adapt in response to perceived inability to satisfy demand.

## Introduction

It has long been known that manipulating an organism’s diet can profoundly impact its behavior, physiology, and most notably, its lifespan. Since the early 1900s, studies in both rodents (McCay et al., 1935; Weindruch and Walford, 1982; Weindruch et al., 1986) and fruit flies (Chippindale et al., 1993) have shown that reductions in total calorie consumption significantly extend lifespan. Since then, this method of dietary restriction has also been shown to extend lifespan of worms (Lakowski and Hekimi, 1998), fish (Masoro, 2002), rodents (Masoro, 2002), dogs (Masoro, 2002), and non-human primates (Mattison et al., 2017; Pifferi et al., 2018), indicating highly conserved roles for food as a modulator of aging. Additional studies have since found that reduction in specific macronutrients (i.e., protein or carbohydrates) is also sufficient to extend lifespan in many model systems. Many animals, including primates (Raubenheimer et al., 2015; Uwimbabazi et al., 2021), consume to specific nutrient ratios that maximize overall fitness. Further, geometric frameworks for nutrition studies have shown that low protein-high carbohydrate diets maximize lifespan in flies (Lee Kwang et al., 2008; Bruce et al., 2013) and mice (Solon-Biet Samantha et al., 2014) compared to calorically equivalent high protein-low carbohydrate diets, emphasizing the importance of specific nutrients, rather than total calories, in the context of aging.

Much of the literature on the mechanisms by which calorie or nutrient restriction influences health and longevity has focused on the activity of major metabolic integrator systems in peripheral tissues, such as mTOR (mammalian target of rapamycin) (Selman et al., 2008; Harrison et al., 2009), insulin signaling (Clancy et al., 2001; Hwangbo et al., 2004), and AMPK (Anisimov et al., 2005; Burkewitz et al., 2015); altered activity of these systems as a result of lower food consumption often leads to increased longevity (Burkewitz et al., 2012; Pan and Finkel, 2017). However, many methods of dietary manipulations influence lifespan independent of changes in consumption (Mair et al., 2005; Linford et al., 2011), suggesting that, in addition to canonical peripheral tissue integrator systems, the interpretation of nutrient sensing in the brain is also an important modulator of lifespan.

Indeed, a growing body of evidence suggests that perception of environmental cues by the nervous system plays an important role in healthy aging, and manipulation of sensory pathways is a robust modulator of lifespan. Ablation of specific sensory neurons is sufficient to extend lifespan in *C. elegans* (Apfeld and Kenyon, 1999), and sensing hypoxic conditions and cold temperature do so as well (Xiao et al., 2013; Leiser et al., 2015). In flies, loss of olfactory and taste perception extends lifespan (Libert et al., 2007; Ostojic et al., 2014; Waterson et al., 2014), and in mice, loss of pain perception increases lifespan, as does olfactory perception of same-sex pheromones (Riera Céline et al., 2014; Riera et al., 2017), suggesting the role of the central nervous system as a global regulator of overall physiology and lifespan is highly conserved.

In at least some cases, the mechanisms by which environmental perception influences aging rely on evolutionarily conserved neuromodulators, such as serotonin, particularly in regard to nutrient perception. When flies are presented with the opportunity to construct their own diet as opposed to the standard homogenous nutrient mixture, lifespan is shortened in a manner dependent on neuronal signaling through the 5-HT2A serotonin receptor (Ro et al., 2016; Lyu et al., 2021). In *C. elegans*, the lifespan extension *via* dietary restriction is reduced when worms can smell, but not access, food, and this effect requires both dopamine and serotonin signaling (Zhang et al., 2021; Miller et al., 2022).

Despite the clear links between 5-HT2A and aging in specific nutritional contexts, little is known about whether it alters lifespan in non-stressful or nutrient-replete conditions, and, if so, the mechanisms by which this occurs. Here, we report that loss of 5-HT2A extends lifespan in protein replete conditions and induces a resistance to changes in dietary protein levels that normally modulate lifespan, suggesting that 5-HT2A is important for mediating behavioral and physiological adaptations to protein availability in the environment. We further show that 5-HT2A^*−/−*^ null mutant flies exhibit consistently lower protein consumption and that activation of specific 5-HT2A^*+*^ neurons induces protein feeding. Therefore, serotonin signaling through receptor 5-HT2A is likely recruited in response to physiological protein demand to promote motivation for protein intake, and chronic reduction of protein-drive through loss of 5-HT2A signaling leads to a lower physiological protein target which alters protein metabolism and utilization in a way that is favorable for lifespan.

## Methods

### Contact for reagent and resource sharing

Further information and requests for resources and reagents should be directed to and will be fulfilled by the corresponding author, SP (spletch@umich.edu).

### Fly stocks and husbandry

The w-; CS stock was established by mixing a population of *w* ^*1118*^ flies with standard Canton-S (CS) flies for more than 10 generations and re-isolating white-eyed flies. The 5-HT2A^*−/−*^ null mutants containing a *GAL4* element in place of the first coding exon (Qian et al., 2017) were generously donated by Yi Rao, Peking University. The broad-expressing *5-HT2A-GAL4* was created by replacing a MiMIC insertion in the *5-HT2A* locus with a *GAL4* element (Gnerer et al., 2015) and was kindly shared by Herman Dierick, Baylor College of Medicine. The restricted *5-HT2A-GAL4* expressing in the superior medial protocerebrum (BDSC #38744) contains a *GAL4* element in the promoter region of *5-HT2A*. *Npf-GAL4* contains a *GAL4* element fused to the regulatory sequence region for *npf* (BDSC# 25682). Neuronal activation experiments were performed using*; UAS-CsChrimson* (BDSC #55135), which was back-crossed to the w-; CS control stock for 10 generations. Neuronal inhibition experiments were performed using the anion channelrhodopsin (*GtACR1*) fused to a *UAS* element (Mauss et al., 2017), which was generously shared by Monica Dus, University of Michigan and back-crossed to the w-; CS control stock for 10 generations.

All fly stocks were maintained on a standard cornmeal-based larval growth medium (produced by LabScientific Inc. and purchased from Fisher Scientific) in a constant environment (25°C, 60% humidity) with a 12:12 h light:dark cycle. We controlled the developmental larval density by aliquoting 32 µl of collected eggs into individual bottles containing 25 ml of food. Following eclosion, mixed-sex flies were kept on SY10 (10% [w/v] sucrose and 10% [w/v] yeast) medium for 2–3 days until they were used for experiments. Pioneer table sugar (purchased from Gordon Food Service, MI) and MP Biomedicals Brewer’s Yeast (purchased from Fisher Scientific) were used in our study.

### Diet compositions

See table below for information on each diet used in lifespan and/or ConEx experiments (values are based on 1 L total volume):

**Table:** 

	SY10	S15Y5	S5Y15	S10Y5	S10Y15	S10Y20	S5	S10	S20
Agar (g)	20	20	20	20	20	20	20	20	20
Sucrose (g)	100	150	50	100	100	100	50	100	200
Yeast (g)	100	50	150	50	150	200			
Tegosept (20%)	15	15	15	15	15	15			
Propionic acid (ml)	3	3	3	3	3	3			

### qPCR

All flies for qPCR analysis were flash frozen in liquid nitrogen for storage at −80^°^C. Total mRNA was extracted in FastPrep Lysing Matrix D tubes (MP Biomedicals) using cold TRIzol (ThermoFisher Scienctific, Inc.) according to the manufacturer’s directions. The RNA-containing fraction was then removed by pipet to a fresh, autoclaved microcentrifuge tube for RNA precipitation using 100% ethanol. After 30 min at −80^°^C, the RNA pellet was collected by spinning at 13,000 rpm in a benchtop centrifuge. The pellet was washed 2X using 70% ethanol in DEPC water and then dissolved in 20 μl DEPC water for spectrophotometer analysis at 260 nm. Equal amounts of RNA from each sample were then subjected to reverse transcription using SuperScript III RT enzyme and protocol (Invitrogen) according to the manufacturer’s directions. The subsequent cDNA was then subjected to qPCR analysis in a StepOne Plus 96-well Thermocycler (Applied Biosystems) using SYBR Green PCR Master Mix (Applied Biosystems) according to the manufacturer’s instructions. The primer sequences used for amplification of the *5-HT2A* cDNA are as follows:

Forward primer: 5′ TCG​CCA​GCC​GTT​TAT​TGA​CT 3′

Reverse primer: 5′ CTC​CGC​TTC​GTC​GAT​AGC​TT 3′

### Metabolic assays

After the flies were aged on SY10 for 10–14 days (food was changed every 2–3 days), experimental flies were quickly frozen, collected into groups of five, and then homogenized in 200 μl of ice-cold phosphate-buffered saline containing 0.1% Triton X-100 for 30 s at 30 Hz using a QIAGEN TissueLyser. For triacylglycerol (TAG) quantification, the homogenate (20 μl) was added into 200 μl of Infinity Triglyceride Reagent (Thermo Electron Corp.) and incubated at 37°C for 10 min with constant agitation. TAG concentrations were determined by measuring the absorbance at 520 nm and estimated by a known triglyceride standard. For protein measurement, 5 μl of fly homogenate was incubated with 200 μl of (1:50) 4% (w/v) cupric sulfate/bicinchoninic acid solution (Novagen) at room temperature for 30 min. For protein excretion measurements, flies were fed SY10 food for 72 h (20 flies per vial, 7-8 replicates for each group). The food was then removed from the vials and 1 ml of Milli-Q water was used to wash the excrement from the vials. This solution was then passed through a dechorionation sieve to remove any eggs and 10 μl of the wash volume was used to measure protein concentration. Protein concentrations were calculated by measuring the absorbance at 562 nm through the comparison with bovine serum albumin standards for all protein assays. For mass measurements, flies were aged on SY10 for 10–14 days, then separated into Eppendorf tubes for weight measurements. The tubes were kept at −20°C for 30 min to ensure the flies were not moving prior to wet mass measurements. The tubes were then placed in a 37°C oven for 3 days before measuring dry mass. Average weight, TAG, and protein values were based on at least 10 independent biological replicates from multiple vials.

### Reproduction assays

Virgin flies of each genotype were collected and mated to one week-old Canton-S males in a 1:1 male to female ratio (five females and five males were co-housed in SY10 vials with ten vials per treatment). Exposure to males began 48 h prior to the start of the experiment. Vials were flipped daily, and the total number of eggs were counted each day then summed for each vial over 7 days.

### Consumption-excretion assays

We used the protocol described previously (Shell et al., 2018). Briefly, experimental flies were co-housed in vials (10 flies per vial, 8–10 replicates for each group) for 2–3 days following eclosion and then sorted into individual sex cohorts on SY10 food. After 10–14 days, the flies were transferred onto diets with 1% (w/v) FD and C Blue No. 1 in varying percentages from 5% to 20% of Yeast or Sucrose (w/v) based on the experiment (see table below under Diet Compositions for details). Vials were discarded if one or more dead flies were observed after the 24 h feeding period. For ConEx experiments coupled to optogenetics, 3 min of 40 Hz red light was supplied every 15 min to minimize depolarization block (Shao et al., 2017). Excreted dye (ExVial) was collected by adding 3 ml of Milli-Q water to each vial followed by vortexing for 10 s. The concentration of the ExVial dye in water extracts was determined by reading the absorbance at 630 nm, which was used to infer macronutrient consumption.

### Opto fly liquid-food interaction counter

Flies were tested on the Fly Liquid-Food Interaction Counter (FLIC) system as previously described to monitor feeding behaviors (Ro et al., 2014) coupled to optogenetic activation technology. Specifically, custom lids were produced containing one LED for each well in the FLIC chamber, allowing precise control over the dynamics of the light timing, duration, and frequency. Easycargo Aluminium Radiator Cooler Heat Sinks were used to dissipate any heat generated by the LEDs [DigiKey manufacturer numbers: LXM2-PD01-0050 (red), LXML-PM01-0100 (green)]. Each liquid-food reservoir contained either 2% (w/v) yeast extract +1% (w/v) sucrose or 1% (w/v) sucrose in 1% Tegosept (v/v) and 4 mg/l MgCl_2_. For closed-loop experiments, 100 µM denatonium in 1% Tegosept (v/v) and 4 mg/l MgCl_2_ was used. Flies were anesthetized briefly on ice and manually aspirated into the *Drosophila* feeding monitors (DFMs). Each DFM was loaded with flies from at least two treatment groups to reduce technical bias from the DFM signals. LED lights were always pulsed at 40 Hz with a 32% duty cycle. In the open-loop activation experiments, red light was provided 12 s every six minutes for experiments using the broad *5-HT2A-GAL4* driver and for three minutes every fifteen minutes for the more restricted *5-HT2A-GAL4* (BDSC #38744). For open-loop inhibition experiments, green light was supplied for three minutes every fifteen minutes. Red light was pulsed when flies interacted with the liquid food in closed-loop experiments. All LEDs were pulsed at 40 Hz to minimize depolarization block (Shao et al., 2017). For closed-loop experiments, red light was pulsed at 40 Hz upon interaction with the liquid food well and included a one second decay after food interaction ceased. Food interactions were analyzed using custom R code, which is available on GitHub at https://github.com/PletcherLab/FLIC_R_Code. Default thresholds were used for analysis. Flies that had zero feeding events over the testing interval were removed from the analysis.

### Neuronal activity experiments

Flies emerged on CT food and were collected after 48 h to allow for mating. Female flies were then separated from males and placed in individual vials during nutrient exposure to minimize potential neuronal signals from social cues. Vials were prepared containing 2% agar coated with 200 mM sucrose ±2.5x complete amino acid solution, prepared as previously described (Piper et al., 2014) Nutrient exposure time was 48 h, after which the female brains were dissected and imaged as described below using a wavelength of 488 nm to visualize the NFAT GFP signal.

### Brain dissection

Four days post-eclosion brains from adult females exposed to 200 mM sucrose ±2.5x complete amino acid solution conditions were dissected in ice-cold phosphate buffer saline (PBS) using sharpened tweezers and fixed in PBS containing 4% paraformaldehyde for 60 min at room temperature. The brains were washed thoroughly with 1 ml of PBS with 0.1% Triton-X (PBS-T), then moved using a wide-bore pipet tip to a glass slide with Vectashield (Vector Laboratories) and sealed using a coverslip and clear nail polish for immediate imaging.

### Imaging and analysis

Imaging was carried out using an Olympus FLUOVIEW FV3000 confocal microscope. The brains were brought into focus with 10x (0.40 NA) objective lens before switching to 20x (0.75 NA). Extra care was taken in order not to saturate the image. Images were acquired at 1,024 × 1,024 pixels with a step size of 3.0 micron. The laser power and the parameters for image acquisition was kept the same between control and treatment groups.

For data analysis, the imaging files were analyzed in the publicly available imaging software, Fiji. Selective slices were combined and collapsed into a single image using SUM slices. Brightness and contrast were adjusted manually when required for better visualization of the image. Background was calculated from the brain region adjacent to the ROI and subtracted. Representative images are displayed in mpl-inferno pseudo color.

### Survival assays

Lifespans were measured using established protocols (Linford et al., 2013). Unless otherwise noted, 10 replicate vials (∼200 experimental flies) were established for each treatment. Flies were transferred to fresh media every 2–3 days, at which time dead flies were removed and recorded using the DLife system developed in the Pletcher Laboratory (Linford et al., 2013). Flies were kept in constant temperature (25°C) and humidity (60%) conditions with a 12:12 h light:dark cycle. For optogenetic lifespans, flies were kept in specially designed rigs containing red or green LEDs, to activate or inhibit neurons, respectively. Controls were kept in a dark box in the same incubator and flipped under dim red light. Red LEDs were pulsed at 2 Hz for activation experiments and green LEDs were pulsed at 40 Hz for inhibition experiments.

### Statistics

Unless otherwise indicated, pairwise comparisons between different treatment survivorship curves were carried out using the statistical package R within DLife (Linford et al., 2013). Each *p*‐value was obtained using a Log‐Rank analysis. For testing the interaction between genotypes and diets, we used Cox-regression analysis to report *p*-value for the interaction term. To test the effects of diet and genotype involved in food consumption, we performed Two-way ANOVA followed by a Bonferroni post-hoc significance test or ANCOVA. For OptoFLIC data, total licks for a given time period were fourth-root transformed, which was empirically found to normalize the majority of FLIC data. Significance was determined using a one-sided *t*-test. Significance for metabolic assays was measured using a two-sided *t*-test. For imaging experiments, a one-sided *t*-test was used to determine significance.

## Results

### Loss of the serotonin receptor 5-HT2A extends lifespan

**FIGURE 1:**
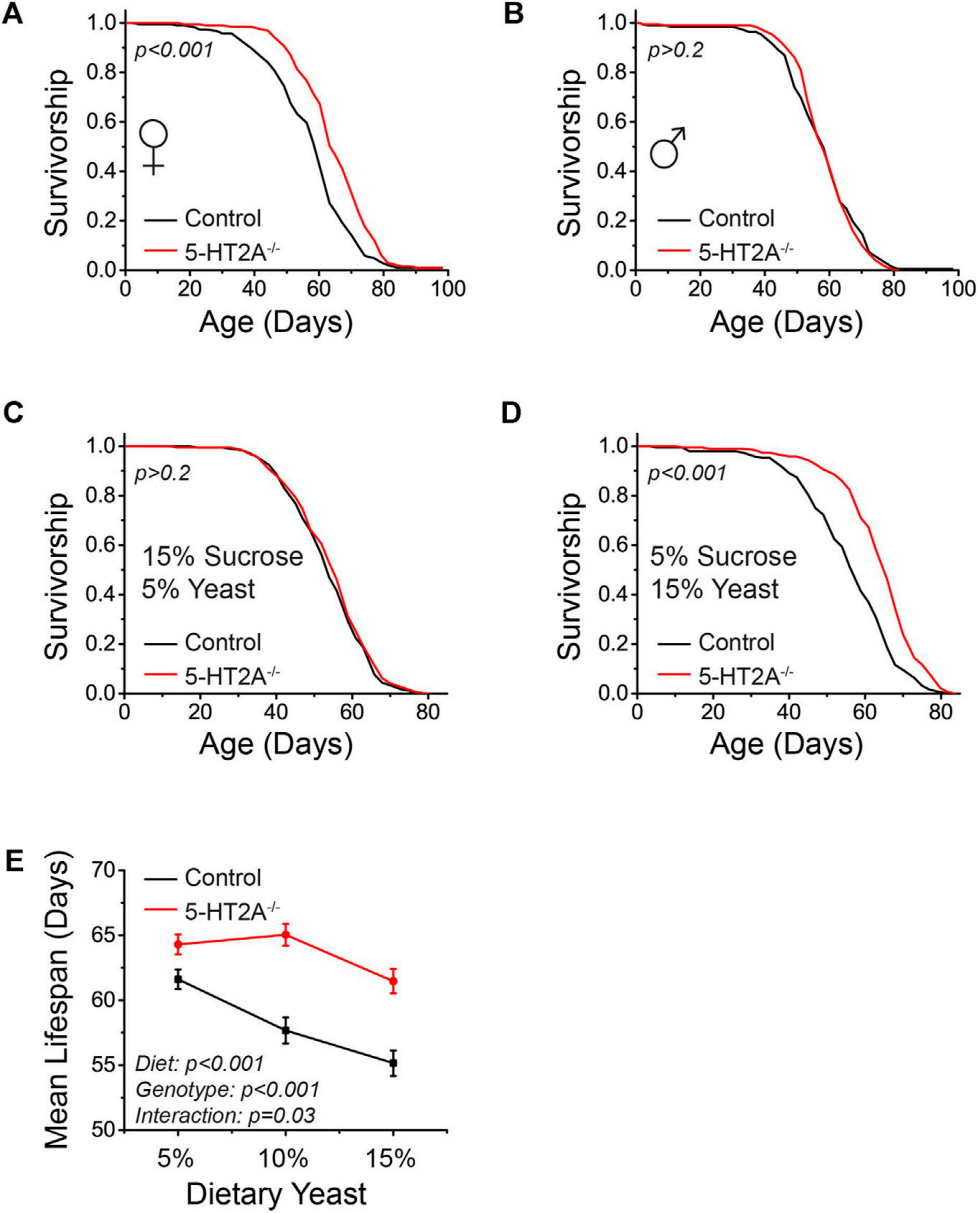
5-HT2A interacts with protein levels to modulate lifespan. **(A)** Female *5-HT2A* ^*−/−*^ mutants are long-lived relative to white-eyed Canton-S (w-; CS) controls on a standard laboratory diet consisting of 10% sucrose and 10% yeast (*n* = 193 and 188, log-rank analysis *p* < 0.001). **(B)** Male *5-HT2A* ^*−/−*^ mutants are not long-lived relative to w-; CS controls on a 10% sucrose and 10% yeast diet (*n* = 194 and 192, log-rank analysis *p* = 0.71). **(C)** *5-HT2A* ^*−/−*^ mutant females do not show a lifespan extension on a 15% sucrose/5% yeast diet (*n* = 193 and 185, log-rank analysis *p* = 0.4). **(D)** *5-HT2A* ^*−/−*^ mutant females are long-lived on a 5% sucrose/15% yeast diet (*n* = 195 and 193, log-rank analysis *p* < 0.001). **(E)** The mean lifespan of w-; CS control females decreases significantly as dietary protein increases (*n* = 187–195, One-way ANOVA Diet: *p* < 0.001), and dietary protein slightly significantly affects lifespan in *5-HT2A* ^*−/−*^ mutant females (*n* = 187–195, One-way ANOVA Diet: *p* = 0.01). The *5-HT2A* ^*−/−*^ mutant lifespan is significantly different from that of controls across diets containing 5%–15% yeast (*n* = 187–195, ANCOVA Diet: *p* < 0.001 Genotype: *p* < 0.001 Interaction: *p* = 0.03). Censored observations were ignored for the analysis of mean longevity.

To better understand the role of serotonin receptor 5-HT2A in aging, we studied flies homozygous for a putative null mutation (*5-HT2A* ^*−/−*^), which contained a *GAL4* element in place of the first coding exon (Qian et al., 2017). The *5-HT2A* ^*−/−*^ line used in these studies is a verified mutant used in other published studies and we confirmed that it lacked measurable *5-HT2A* mRNA by quantitative PCR (Gasque et al., 2013; Howard et al., 2019). We first asked whether *5-HT2A* ^*−/−*^ mutant flies were long-lived when aged on a standard laboratory diet consisting of 10% sucrose and 10% yeast (w/v, SY10) relative to a laboratory control strain (Canton-S) to which it had been extensively backcrossed. We found that *5-HT2A* ^*−/−*^ mutant females lived significantly longer than control flies (Figure 1A). Loss of 5-HT2A did not have a significant effect on male lifespan (Figure 1B), leading us to focus on females for the majority of the study. Previous experiments had shown that 5-HT2A is required for modulating fly lifespan when the major dietary components of sucrose and yeast are presented separately, compared to when they are mixed and presented as a single, homogeneous mixture (Ro et al., 2016). We therefore wondered whether altering the levels of sucrose or yeast in our fixed diet would influence the magnitude of the mutant lifespan phenotype. To examine this, we measured the lifespans of *5-HT2A* ^*−/−*^ mutant and control females on both yeast- and sucrose-rich diets, consisting of 15% yeast/5% sucrose or 5% yeast/15% sucrose, respectively. We observed that *5-HT2A* ^*−/−*^ mutants were only long-lived on the yeast-rich diet and showed no difference in lifespan on the 5% yeast/15% sucrose diet (Figures 1C,D), suggesting that either a low sugar or high yeast environment promotes extended lifespan in *5-HT2A* ^*−/−*^ mutant flies.

Dietary protein as yeast is particularly impactful in fly aging, as it largely drives the dietary restriction (DR) lifespan extension phenotype (Chippindale et al., 2004; Min and Tatar, 2006). Decreasing the levels of protein typically increases lifespan in a dose-dependent manner, up to a point where protein levels are presumably too low to support essential metabolism (Min and Tatar, 2006; McCracken et al., 2020), and we hypothesized that loss of 5-HT2A might alter the magnitude of this response. Holding sucrose levels constant, we therefore titrated the amount of protein in the diet and measured the lifespan of *5-HT2A* ^*−/−*^ mutant and control flies. Not unexpectedly, the mean lifespan of control animals increased significantly as dietary protein levels decreased (Figure 1E). The mean lifespan of *5-HT2A* ^*−/−*^ mutant flies, however, was less affected by dietary protein and remained high even in protein-rich conditions (Figure 1E). There was also a statistically significant interaction between genotype and diet (*p* = 0.03), supporting the conclusion that the mean lifespans of the two genotypes respond differently to diet. This suggests that loss of 5-HT2A alters the response to protein, potentially making flies more resistant to reductions in lifespan that normally accompany increases in dietary protein.

### 5-HT2A influences yeast consumption

**FIGURE 2:**
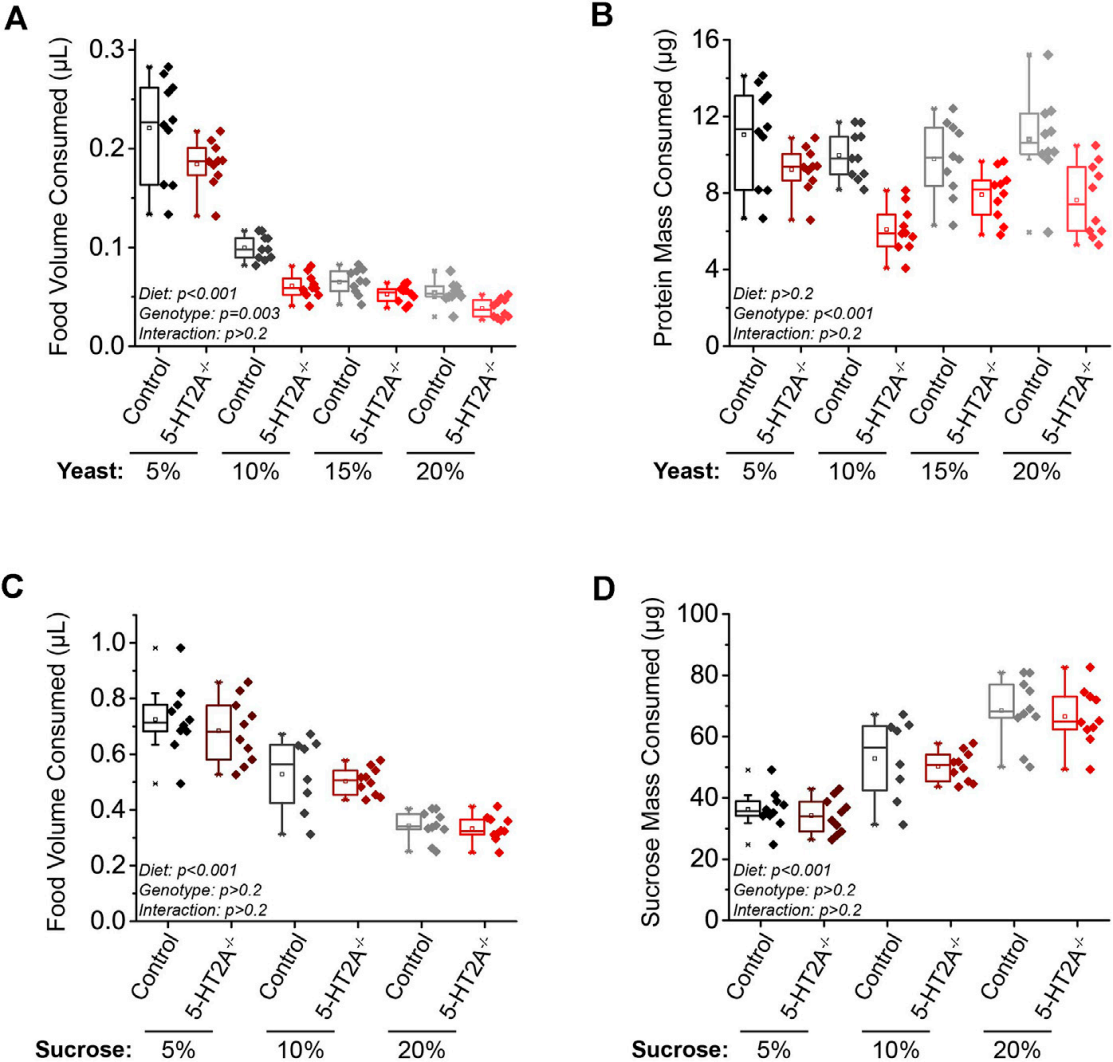
5-HT2A modulates the protein consumption set-point. **(A)** As dietary yeast increases, both *5-HT2A* ^*−/−*^ mutants and w-; CS controls consume less (*n* = 10 where each replicate is comprised of 10 flies, ANCOVA Diet: *p* < 0.001 Genotype: *p* = 0.003 Interaction: *p* = 0.242), and when these data are scaled to represent the mass of protein consumed across diets, **(B)** *5-HT2A* ^*−/−*^ mutants show a decreased protein consumption target across diets (*n* = 10 where each replicate is comprised of 10 flies, ANCOVA Diet: *p* = 0.322 Genotype: *p* < 0.001 Interaction: *p* = 0.605). Relative to controls, *5-HT2A* ^*−/−*^ mutants show no differences from controls in the **(C)** total volume (*n* = 8–10 where each replicate is comprised of 10 flies, ANCOVA Diet: *p* < 0.001 Genotype: *p* = 0.263 Interaction: *p* = 0.594) or **(D)** mass of sucrose consumed across three different concentrations of a sucrose-only diet (*n* = 8–10 where each replicate is comprised of 10 flies, ANCOVA Diet: *p* < 0.001 Genotype: *p* = 0.409 Interaction: *p* = 0.996). All experiments were conducted in females.

To further examine the interaction between the effects of 5-HT2A loss and dietary yeast, we investigated its role in feeding behavior. We titrated levels of yeast in fly diets and estimated food consumption in *5-HT2A* ^*−/−*^ mutant and background control flies by spiking diets with a non-metabolizable blue dye (FD&C Blue 1) and used the amount of excreted dye as an estimate of consumption [i.e., the ConEx assay (Shell et al., 2018)]. Using the standard 10% sugar/yeast laboratory medium used in lifespan measurements as a reference diet, we measured consumption on diets containing 10% sucrose and ranging from 5% to 20% yeast (w/v). In flies from both genotypes, we observed that the volume of food consumed decreased as the amount of yeast in the diet increased (Figure 2A). When presenting these data in terms of the mass of yeast consumed, we observed a significant effect of genotype and no significant effect of dietary yeast concentration, supporting the notion that flies have a protein consumption target, ie., the amount of protein voluntarily ingested that we speculate satisfies their needs, driving their feeding behavior in fixed diets (Figure 2B) (Lee Kwang et al., 2008). The genotype effect was indicative of a consistent reduction of protein consumption in *5-HT2A* ^*−/−*^ mutant flies, suggesting the possibility that they exhibit a lower protein consumption target. Differences in the mass of sucrose consumed matched the pattern of total consumption because sucrose was held constant in these diets (Supplementary Figure S1A). Similar to lifespan, loss of *5-HT2A* ^*−/−*^ did not significantly affect feeding in males. Both mutant and control genotypes responded similarly to increased dietary protein by consuming less, and there was no significant effect of diet or genotype on the mass of yeast consumed (Suppulementary Figures S1B,C). Similar to females, sucrose mass consumption decreased as dietary protein increased, but there was no effect of genotype in males (Supplementary Figure S1D).

A similar experiment in which feeding was measured on diets ranging from 5% to 20% sucrose (w/v) only (to avoid confounding effects from yeast) revealed that female flies of both genotypes decreased total volume consumed as the concentration of dietary sucrose increased (Figure 2C). However, when scaled to present the mass of sucrose consumed, female flies of both genotypes consumed a higher mass of sucrose as concentration increased (Figure 2D). Unlike the experiments in which we manipulated dietary yeast, however, we observed no significant difference in pattern of consumption between *5-HT2A* ^*−/−*^ mutant and control flies on sucrose diets (Figures 2C,D).

### 5-HT2A modulates protein body content

**FIGURE 3:**
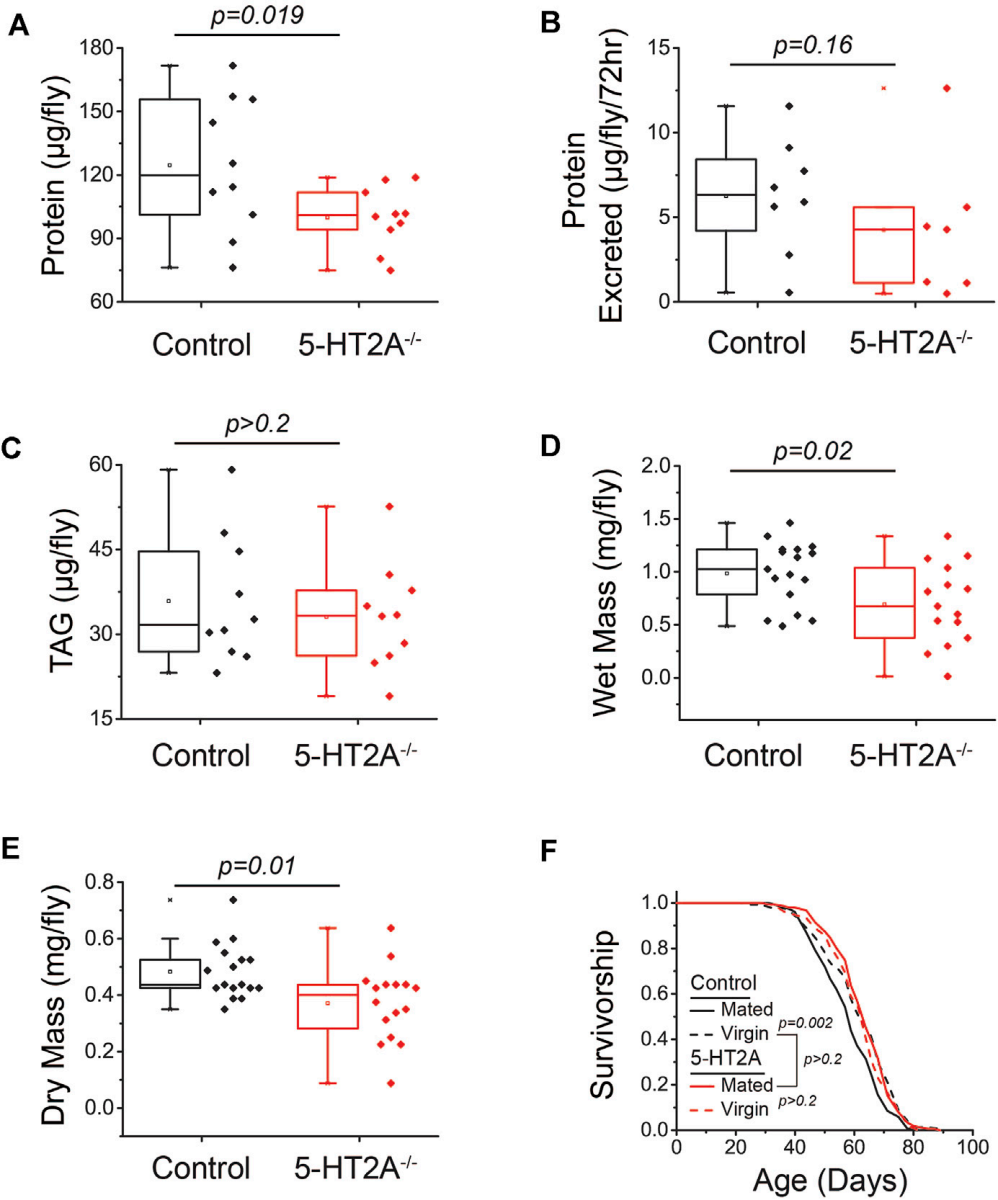
5-HT2A modulates protein body content. **(A)** *5-HT2A* ^*−/−*^ mutants show reduced protein levels relative to controls, as measured by BCA (*n* = 10 where each replicate is comprised of 5 flies, one-sided *t*-test *p* = 0.019) **(B)** but excrete the same levels of protein as controls (*n* = 7 and 8 where each replicate is comprised of 20 flies, one-sided *t*-test *p* = 0.16). **(C)** *5-HT2A* ^*−/−*^ mutants show no differences in triglyceride (TAG) levels relative to controls (*n* = 10 where each replicate is comprised of 5 flies, two-sided *t*-test *p* = 0.56). *5-HT2A* ^*−/−*^ mutants show a significant reduction in **(D)** wet mass (*n* = 15–17 where each replicate is comprised of 8 flies, two-sided *t*-test *p* = 0.02) and **(E)** dry mass relative to controls (two-sided *t*-test *p* = 0.01). **(F)** Lifespan of control flies is shortened by mating (*n* = 153 and 146, log-rank analysis *p* = 0.002), while the and the lifespan of *5-HT2A* ^*−/−*^ mutants is not (*n* = 151 and 134, log-rank analysis *p* = 0.3) and is not significantly different from control virgin lifespan (*n* = 151 and 146, log-rank analysis *p* = 0.9). All experiments were conducted in females.

A reduced protein consumption target in *5-HT2A* ^*−/−*^ mutants might be reflected in lower protein content in the body. Indeed, we found that *5-HT2A* ^*−/−*^ mutant females exhibited decreased whole-body protein content (Figure 3A). To evaluate if nutrient absorption was involved in female body composition differences, we measured protein content in their excretion and observed no differences between *5-HT2A* ^*−/−*^ mutant and control females (Figure 3B). We also asked whether changes in body composition in *5-HT2A* ^*−/−*^ mutant females was specific to protein or whether it extended to other forms of nutrient storage. In *Drosophila*, excess carbohydrates are converted and stored primarily as triglycerides (TAG), and we found no differences in female TAG levels (Figure 3C). To determine whether the reduction in total protein influenced overall body mass, we measured both wet and dry mass and observed a significant reduction in both measures in *5-HT2A* ^*−/−*^ mutant females (Figures 3D,E). The reduction in body mass is likely due to lower protein levels in *5-HT2A* ^*−/−*^ mutants, with the lack of differences in TAG suggesting fat storage levels and carbohydrate usage are similar.

### Loss of 5-HT2A mimics lifespan extension in virgin flies

Our behavioral and physiological data led us to speculate that the absence of 5-HT2A receptor results in a chronic state of perceived protein limitation, independent of its availability in the diet. This situation might be expected to stimulate adaptive processes that reduce protein utilization, which would manifest as increased lifespan, particularly when dietary protein is replete. In this view, *5-HT2A* ^*−/−*^ mutant flies would exhibit lifespan phenotypes that are associated with physiological states of low protein utilization and would be resistant to manipulations that subsequently increase it. Female reproductive status is associated with such a state; virgin female flies lack developing embryos, exhibit a reduced drive for protein consumption, and are long-lived (Ro et al., 2014). Mating increases reproductive output and drives protein feeding. We found that as little as three days of mating following eclosion (following our typical lifespan measurement protocol) significantly reduced the lifespan of control flies fed our standard 10% sugar-yeast diet (Figure 3F). *5-HT2A* ^*−/−*^ mutant flies, however, were long-lived regardless of mating status; average mutant lifespan was not influenced by mating and was not significantly different from that of the control genotype virgin flies (Figure 3F). As postulated by Ro et al. (2016), several lines of evidence support the notion that extended lifespan in mutant females is independent of reduced nutrient intake and likely reflects a defect in normal physiological responses to perceived protein availability. First, feeding differences did not depend on mating status (Supplementary Figure S2A). Second, mutant females showed no differences in egg-laying relative to control flies (Supplementary Figure S2B). Third, the lifespan extension of *5-HT2A* ^*−/−*^ mutants persisted in the absence of protein in the diet, eliminating differences in protein consumption as a required component of the lifespan extension (Supplementary Figure S2C).

### Activation of *5-HT2A* ^*+*^ neurons promotes interaction with protein

We next sought to investigate whether 5-HT2A signaling influences motivational or reward circuitry related to protein consumption, which would affect responses to perceived availability. Published data suggest that 5-HT2A signaling is not involved in specifying protein demand but instead influences protein feeding by perhaps 1) stimulating its consumption or 2) by acting subsequent to consumption to reinforce feeding (Ro et al., 2016). The first model predicts that activation of 5-HT2A neurons would precede protein feeding and that neuronal activation would promote protein, but not carbohydrate, feeding and would not be generally rewarding. On the other hand, if 5-HT2A were acting to reinforce protein feeding, we might expect that activation of 5-HT2A neurons would follow protein feeding and that their activation would reinforce behavior when closely paired with feeding events.

**FIGURE 4:**
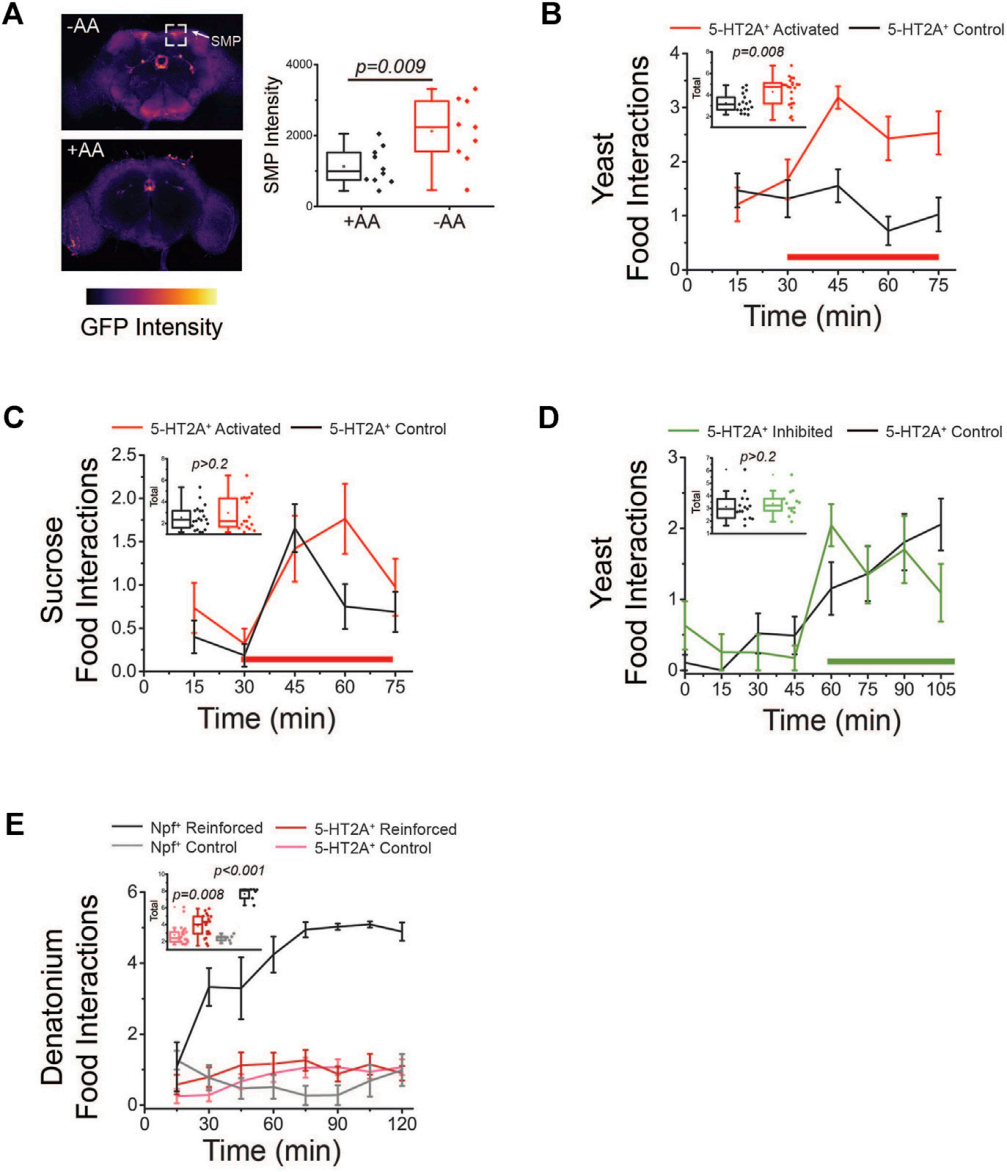
Activation of *5-HT2A* ^*+*^ neurons promotes protein feeding behaviors. **(A)** Amino acid (AA) deprivation activates *5-HT2A* ^*+*^ neurons in the superior medial protocerebrum (SMP, top) relative to amino acid replete controls (bottom). Quantification of SMP intensity (*n* = 10 and 9, two-sided *t*-test *p* = 0.009). **(B)** Activation of *5-HT2A* ^*+*^ neurons (red bar indicates red light activation period) promotes acute interaction with a protein solution. Inset: Quantification of total interactions during the light period (*n* = 15 and 22, one-sided *t*-test *p* = 0.008). **(C)** Activation of *5-HT2A* ^*+*^ neurons (red bar indicates red light activation period) does not significantly alter interactions with a sucrose-only solution. Inset: Quantification of total interactions during the light period (*n* = 18 and 15, two-sided *t*-test *p* = 0.32). **(D)** Inhibition of *5-HT2A* ^*+*^ neurons (green bar indicates green light inhibition period) does not alter interactions with a protein solution. Inset: Quantification of total interactions during the light period (*n* = 15 and 17, two-sided *t*-test *p* = 0.5). **(E)** Closed-loop activation (red bar indicates red light reinforcement period) of *5-HT2A* ^*+*^ neurons modestly increases interactions with a denatonium solution compared to activation of *Npf* ^*+*^ neurons. Inset: Quantification of total interactions for closed-loop activation of *Npf* ^*+*^ neurons (*n* = 6, two-sided *t*-test *p* < 0.001) and *5-HT2A* ^*+*^ neurons (*n* = 24, two-sided *t*-test *p* = 0.008). All experiments were conducted in females.

To evaluate when and where *5-HT2A* ^*+*^ neuronal activity is responsive to protein availability in food, we used *5-HT2A*-*GAL4* (Gnerer et al., 2015) to express a transcriptional reporter system involving an NFAT-based tracing method (CaLexA) through which sustained neural activity drives expression of GFP (Masuyama et al., 2012). We then quantified fluorescent intensity to identify populations of neurons that showed increased activity upon 48h protein manipulation. Specifically, *5-HT2A* > *NFAT* flies were provided 2% agar coated with 200 mM sucrose +/- 2.5x complete amino acid mix (Piper et al., 2014). Flies fed amino acids exhibited decreased GFP intensity, relative to flies fed sucrose alone, in a population of *5-HT2A* ^*+*^ neurons in the supramedial protocerebrum (SMP; Figure 4A), indicating these neurons were responsive to decreases in dietary amino acids.

We next determined whether acute activation or inhibition of *5-HT2A* ^*+*^ neurons influence how the animals interact with different nutrients. For these studies, we used the Fly Liquid-Food Interaction Counter (FLIC), which measures the precise timing and duration of fly feeding behaviors through the closure of a circuit when a fly inserts its proboscis into a liquid food source (Ro et al., 2014). For neuronal activation, we drove expression of a red light-sensitive channelrhodopsin protein (*CsChrimson*) in all *5-HT2A* ^*+*^ neurons and coupled the FLIC technology to optogenetic stimulation equipment. To accomplish this, we installed red LED lights on top of individual FLIC feeding chambers and executed an open-loop design, whereby light was illuminated for 12 s (40 Hz and 32% duty cycle) every five minutes, activating neurons regardless of the fly’s behavior. We compared feeding interactions between *5-HT2A>CsChrimson* flies and their genotypic controls (see Section 2 for strain details) exposed to either sucrose or yeast food and in the presence or absence of red-light stimulation. When putatively all *5-HT2A* ^*+*^ neurons were activated in this open-loop configuration, we observed a rapid, significant increase in interactions with a protein-containing solution in *5-HT2A>CsChrimson* flies relative to controls, but not with a sucrose-only solution (Figures 4B,C). When these same experiments were conducted in the absence of light, no differences were observed between 5-HT2A-neuronal activated flies and controls in the presence of either protein or sucrose (Supplementary Figures S3A,B). For inhibition, we expressed an inhibitory anion channelrhodopsin (*GtACR1*) in all *5-HT2A* ^*+*^ neurons and substituted green LEDs in an open-loop configuration where green light was pulsed for 3 min every 15 min. This manipulation did not significantly impact interactions with a protein-containing solution (Figure 4D), showing that the activity of *5-HT2A* ^*+*^ neurons does not bi-directionally control protein feeding and suggesting that 5-HT2A signaling is specifically recruited to promote protein feeding behaviors.

To determine whether activation of *5-HT2A* ^*+*^ neurons is sufficient to reinforce behavior regardless of the presence of protein, perhaps as a result of it conveying a more general sense of reward, we exposed flies to a denatonium solution (100 µM), which is bitter and aversive, and activated *5-HT2A* ^*+*^ neurons in response to individual flies’ interaction with it (i.e., a closed-loop configuration). Unlike the robust increase in interactions observed upon activation of putative reward neurons that express the neuropeptide NPF (Shao et al., 2017), we observed only a modest increase in interactions with the bitter solution when *5-HT2A* ^*+*^ neurons were activated (Figure 4E), suggesting *5-HT2A* ^*+*^ neuronal activation alone does not provide a potent reward.

Finally, we also asked whether activation or inhibition of *5-HT2A* ^*+*^ neurons impacted lifespan. We observed that both manipulations significantly shortened lifespan (Supplementary Figures S3C,D). We hesitate to interpret these results in the context of our model because *5-HT2A* ^*+*^ neurons express many receptors and signaling peptides, all of which would be affected by optogenetic manipulations. These effects on lifespan may, therefore, be non-specific and due to the pleiotropic effects of activating or inhibiting a large group of neurons.

In summary, increased neuronal activity was observed when flies were amino acid deprived (Figure 4A), not when then had fed on protein, and *5-HT2A* ^*+*^ neuronal activation was sufficient to drive protein consumption (Figure 4B) but was not generally sufficient to reinforce feeding behavior (Figure 4E). Taken together, therefore, these data support the notion that 5-HT2A signaling is recruited to promote protein consumption, perhaps by establishing a heightened protein consumption target, and to enact a physiological state of higher protein utilization that subsequently accelerates aging.

### Activation of *5-HT2A* ^*+*^ neurons in the supramedial protocerebrum (SMP) promotes protein consumption

**FIGURE 5:**
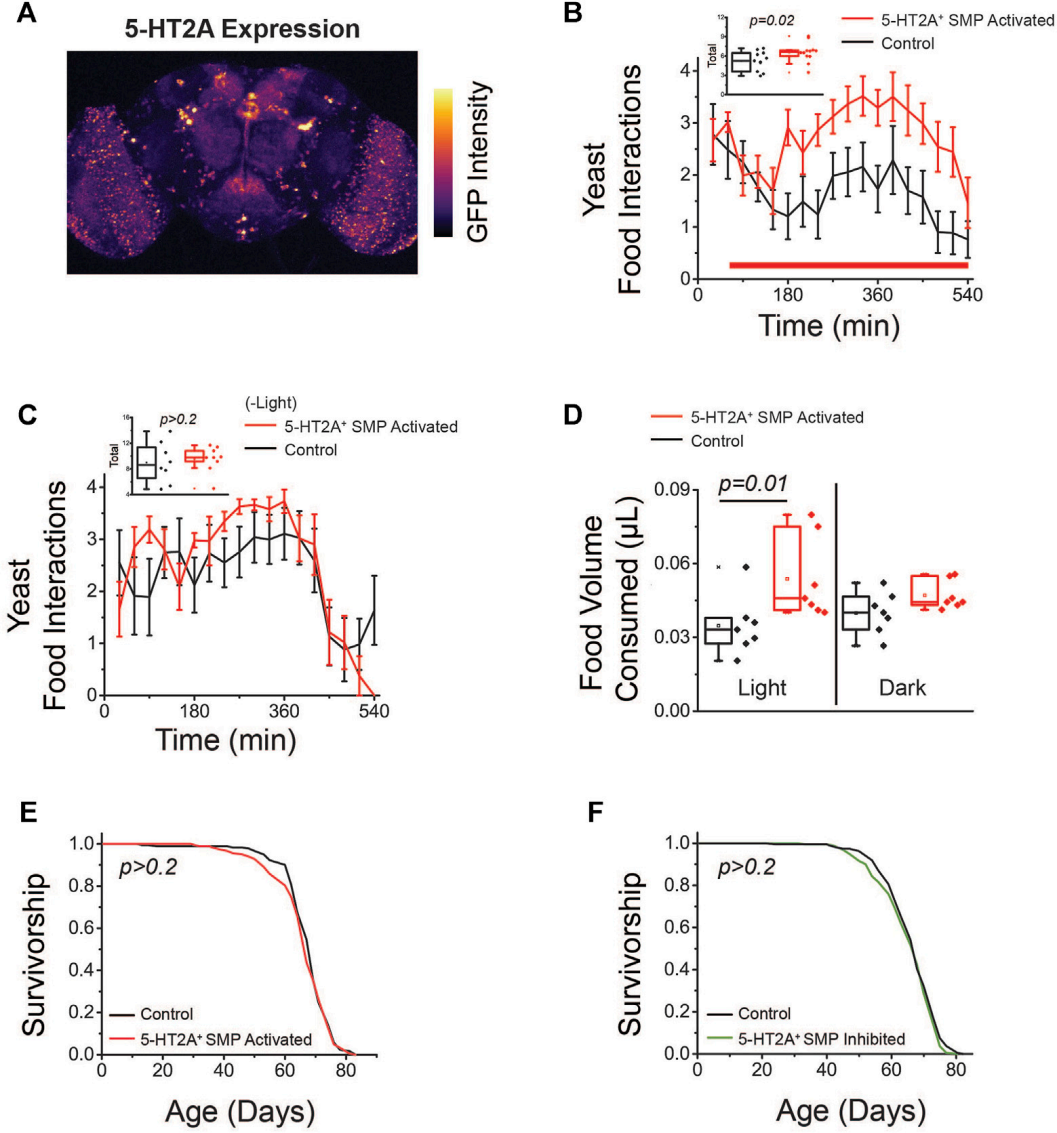
Activation of *5-HT2A* ^*+*^ SMP neurons promotes protein feeding behaviors. **(A)** GFP labeling of neurons driven by *5-HT2A-GAL4* in the brain (image is a max projection). **(B)** Activation of *5-HT2A* ^*+*^ SMP neurons (red bar indicates red light activation period) promotes acute interaction with a protein solution. Inset: Quantification of total interactions during the light period (*n* = 11 and 12, one-sided *t*-test *p* = 0.02). **(C)** Flies expressing *CsChrimson* in *5-HT2A* ^*+*^ SMP neurons do not show differences in interactions with a protein solution in the absence of red light activation (*n* = 8 and 9, two-sided *t*-test *p* = 0.68). **(D)** Activation of *5-HT2A* ^*+*^ SMP neurons promotes consumption of high protein solid food (*n* = 7 where each replicate consists of 10 flies, one-sided *t*-test *p* = 0.01) and flies expressing *CsChrimson* in *5-HT2A* ^*+*^ SMP neurons do not show differences in solid food consumption relative to controls in the absence of red light activation (*n* = 7 where each replicate is comprised of 10 flies, one-sided *t*-test *p* = 0.09). **(E)** Activation (*n* = 182 and 185, log-rank analysis *p* = 0.5) and **(F)** inhibition (*n* = 186 and 191, log-rank analysis *p* = 0.2) of *5-HT2A* ^*+*^ SMP neurons have no effect on lifespan relative to genotypic controls maintained in constant darkness. All experiments were conducted in females.

We next aimed to narrow the population of *5-HT2A* ^*+*^ neurons that influence protein feeding behaviors with the hope of obtaining a better understanding of the relationship between the consumption set point and lifespan. Driving GFP expression with the broad *5-HT2A-GAL4* marks diverse populations of nearly 80 neurons in the *Drosophila* brain (Davie et al., 2018) (Figure 5A). Our neural activity data (Figure 4A) indicated that *5-HT2A* ^*+*^ SMP neurons might influence feeding behavior because the increase in activity upon amino acid deprivation suggested they are responsive to protein demand. We therefore sought to determine whether manipulation of their activity alone might phenocopy the results that we observed following broader 5-HT2A neuronal activation. The FlyLight collection contains thousands of *GAL4* lines that contain fragments of gene-specific promotors (enhancer-trap, or et-*GAL4*), thus, labeling smaller populations of cells that express that gene. One such *GAL4* line for 5-HT2A (R50D04, BDSC #38744) specifically labels SMP neurons and recapitulates the expression of *5-HT2A* ^*+*^ neurons with altered activity in the NFAT data (Jenett et al., 2012). We used this *GAL4* line to express *CsChrimson* and to ask whether optogenetic stimulation was sufficient to increase behavioral interactions with dietary protein. Using a similar open-loop configuration with slightly longer activation periods to ensure against lesser activation of deeper neurons, we observed that activation of R50D04^+^ neurons in the FLIC resulted in a significant increase in interactions with a protein-containing solution relative to controls (Figure 5B), recapitulating the broad 5-HT2A-neuronal activation phenotype. The increase in feeding behaviors was not as rapid as the broad 5-HT2A-neuronal activation phenotype, suggesting the R50D04 line may not provide full coverage over *5-HT2A* ^*+*^ neurons that promote protein feeding behaviors. This difference between genotypes was not observed in the absence of light activation (Figure 5C).

We also asked whether activation of these R50D04^+^ neurons would impact feeding in the same nutritional context as the standard lifespan conditions: a solid food diet consisting of 10% sucrose and 10% yeast (w/v). To test whether activation of *5-HT2A* ^*+*^ SMP neurons also led to increased consumption of solid food, we coupled optogenetic approaches with the ConEx assay, allowing us to activate specific neurons during solid food consumption. In this assay, flies expressing *CsChrimson* in R50D04^+^ neurons were provided with a blue dye spiked diet of 10% sucrose and yeast and housed in vials that were exposed to high-intensity red LEDs. When R50D04^+^ neurons were activated (at 40 Hz and 32% duty cycle) periodically for three minutes every fifteen minutes over 24 h, we saw a significant increase in total consumption relative to the genetic controls, which restored to baseline in a subsequent 24-h dark period (Figure 5D). Both inhibition and activation of these neurons had no effect on lifespan (Figures 5E,F), suggesting protein consumption and lifespan are controlled through, at least partially, distinct mechanisms. Together, these data indicate that this limited subset of *5-HT2A* ^*+*^ neurons in the SMP are critical to promote protein feeding behaviors, and thus, may help encode a protein drive that affects protein feeding behaviors; however, additional *5-HT2A* ^*+*^ neurons may determine how these protein-dependent states impact lifespan.

## Discussion

Our findings support the notion that flies consume to protein targets and that 5-HT2A signaling plays an important role in this. We observed that specific *5-HT2A* ^*+*^ neurons are activated in response to protein deprivation (Figure 4A) and that activation of these neurons promotes protein, but not sucrose, feeding behaviors (Figures 4B,C). This suggests that 5-HT2A signaling does not encode protein demand; rather, upstream serotonergic circuit(s) set demand, and signaling through 5-HT2A promotes consumption of protein. Specific regions of the *Drosophila* brain are strong candidates for circuits in which nutrient demand signals to *5-HT2A* ^*+*^ neurons to promote consumption. The protein deprivation-responsive *5-HT2A* ^*+*^ neurons are found in the superior medial protocerebrum (SMP) and appear to project to a region of the brain known as the fan-shaped body (FSB). The FSB is emerging as a key hub for integrating nutrient cues and information on internal state to make feeding decisions. As a major integration center, it is likely that several upstream circuits that evaluate protein availability converge on the SMP, and may signal through 5-HT2A to satisfy this protein demand. Several neuropeptides (AstA, NPF, and DH44) have been shown to directly inhibit FSB neurons to shift food preferences and dopaminergic neurons indirectly modulate FSB activity through these neuropeptidergic neurons (Sareen et al., 2021). It seems plausible that activity of SMP dopaminergic neurons is regulated by 5-HT2A or that multiple neuromodulator-expressing neurons in the SMP converge on the FSB to influence feeding behaviors. Furthermore, FSB neurons integrate information about internal glucose and fructose levels to induce prolonged feeding when energy levels are low. The activity of these FSB neurons is regulated by glutamatergic signaling *via* neurons in the superior lateral protocerebrum (SLP), which is adjacent to the SMP; however, the inputs for these SLP neurons remain unknown (Musso et al., 2021). As such, it is attractive to speculate that 5-HT2A signaling could also modulate the activity of these glutamatergic neurons, which ultimately converge on the FSB.

Our results also suggest that nutrient set-points are potentially altered by long-term inability to satisfy demand. Long-term discrepancies between nutrient expectation and consumption have been shown to modulate global physiology and lifespan, also in a serotonin-dependent manner. Chronic activation of serotoninergic hunger circuits extends lifespan in flies and leads to a short-term increase in food consumption, which eventually dissipates, putatively as an adaptation to sustained hunger (Weaver et al., 2022). Similarly, relative to diets high in branched-chain amino acids (BCAA)s, flies consuming diets low in BCAAs show higher expression of serotonin synthesis enzymes and serotonin levels in PLP brain neurons, show extended lifespan, and show a short-term increase in feeding that restores to baseline after two weeks (Weaver et al., 2022). The alterations in serotonin levels caused by low BCAA diets was also seen in a recently published mouse study, suggesting that low BCAAs causes these changes using similar mechanisms of action (Solon-Biet et al., 2019). In *C. elegans,* the perception of food cues blunts lifespan extension by dietary restriction and blocking serotonin signaling abrogates this effect (Zhang et al., 2021; Miller et al., 2022). As postulated by Ro et al. (Ro et al., 2016), serotonin-dependent perception of perceived protein limitation modulates lifespan, and the findings from this study suggest that the inability to satisfy protein demand upon loss of 5-HT2A leads to a long-term reduction in protein motivation and may be one mechanism through which these lifespan effects arise.

Given the complexity of serotonin signaling pathways, there are indeed roles for branches of serotonin signaling in various fly cognitive processes in addition to effects on aging. Along with the 5-HT2A receptor studied in this paper, other serotonin receptor pathways participate in learning and memory (Ganguly et al., 2020), mediate depressive-like states (Ries et al., 2017; Hu et al., 2020), and modulate the effects threat perception on physiology (Chakraborty et al., 2019), to provide some examples. Although not directly linked to this work, these studies, together with our own, provide insight into the various neural states influenced by serotonin signaling in the fly. Future work will explore whether changes in fly cognition caused by alterations in serotonin signaling are responsible for the lifespan alteration seen herein, or whether these are separable processes.

Our findings also demonstrate sex-specific differences in both lifespan and protein feeding behaviors; only female flies are long-lived relative to controls (Figures 1A,B) and display a reduced protein consumption target (Figures 2A,B; Supplementary Figures S1A,B). Several possibilities may underlie this sexual dimorphism. First, female flies may be more sensitive to perceived protein limitation, as egg production requires a high level of protein and mating stimulates protein consumption (Ribeiro and Dickson, 2010). As female flies also generally pay a higher cost in reproduction (Partridge, 1989; Sgro and Partridge, 1999), there may, therefore, be higher selective pressures for female behavior and physiology to be responsive to the protein environment relative to males. Second, and not mutually exclusive of the previous point, it is possible that female-specific *5-HT2A* ^*+*^ neurons respond to the perception of the nutritional environment and modulate feeding behavior and lifespan. RNAseq data indicates certain *5-HT2A* ^*+*^ neurons co-express the sex-specific marker, *fruitless* (Janssens et al., 2022), and these neurons may represent interesting candidates for future studies investigating their role in feeding behavior and lifespan.

We note that there is a need for a focused dissection of the populations of *5-HT2A* ^*+*^ neurons that regulate feeding behavior and lifespan. We identified a group of *5-HT2A* ^*+*^ neurons in the SMP that modulate protein feeding behavior (Figure 4A, Figures 5B,D); however, manipulation of the activity of these neurons does not alter lifespan (Figures 5E,F) and is less potent at promoting protein feeding than the population as whole. It remains unknown whether some or all the neurons that modulate feeding also influence lifespan. Additional studies in flies have also implicated 5-HT2A signaling in the modulation of lifespan *via* environmental factors such as exposure to chronic nutrient choice (Ro et al., 2016) or perception of dead conspecifics (Chakraborty et al., 2019), suggesting some still unknown population of *5-HT2A* ^*+*^ neurons may act as important longevity regulators. Examination of neuronal populations that are activated in these scenarios (but not protein deprivation) may help differentiate *5-HT2A* ^*+*^ neurons involved in feeding vs. lifespan. It is also worth noting that 5-HT2A is also expressed in peripheral tissues, such as the salivary gland (Leader et al., 2018), and the role of 5-HT2A signaling in peripheral neurons or other excitable cells in protein feeding behavior and lifespan is an area for further examination.

In nature, organisms must cope with changes in the nutritional environment and optimize behavior and physiology to maximize overall fitness, with responses tailored towards both short-term and long-term adaptions (López-Maury et al., 2008). For instance, in some cases of limited food availability, it would be most adaptive to relocate to find a nutrient-dense food source and activate pathways that promote foraging behavior (Searle et al., 2005; Pretorius et al., 2011). However, in situations of chronic nutrient stress animals must alter their feeding behavior and physiology to survive and reproduce in relatively harsh conditions (Rodgers et al., 2008). Given these fitness consequences, it is likely that adaptive responses to low protein, or perceived low protein, could also apply to other species, even humans. Indeed, specific polymorphisms of the *HTR2A* gene (homologous to *Drosophila 5-HT2A*), are associated with altered nutrient preferences (Prado-Lima et al., 2006), metabolic diseases (Halder et al., 2007), and eating disorders (Genis-Mendoza et al., 2019; Yan et al., 2021). They are also associated with longevity in some populations (Jobim et al., 2008), which raises the interesting possibility that altered 5-HT2A signaling in humans may modulate lifespan, which would make it a potential therapeutic target for aging interventions.

## Data Availability

The original contributions presented in the study are included in the article/Supplementary Material, further inquiries can be directed to the corresponding author.

## Supplementary material

SUPPLEMENTARY FIGURE S1 The 5-HT2A-dependent changes in protein set-point are sexually dimorphic.**(A) **Female *5-HT2A^−/−^* mutant flies show differences in sucrose consumption when 10% sucrose is mixed with varying levels of yeast (*n* = 10 where each replicate is comprised of 10 flies, ANCOVA Diet: *p* < 0.001 Genotype: *p* = 0.003 Interaction: *p* = 0.242). Relative to controls, male *5-HT2A^−/−^* mutants show no differences in **(B)** total consumption (*n* = 9–10 where each replicate is comprised of 10 flies, ANCOVA Diet: *p* < 0.001 Genotype: *p* = 0.675 Interaction: *p* = 0.451) or **(C)** mass of yeast consumed across diets (*n* = 9–10 where each replicate is comprised of 10 flies, ANCOVA Diet: *p* = 0.515 Genotype *p* = 0.206 Interaction: *p* = 0.338). **(D)** Male *5-HT2A^−/−^* mutant flies show differences from controls in sucrose consumption when 10% sucrose is mixed with varying levels of yeast (*n* = 9–10 where each replicate is comprised of 10 flies, ANCOVA Diet: *p* < 0.001 Genotype: *p* = 0.675 Interaction: *p* = 0.451).

SUPPLEMENTARY FIGURE S2 Changes in protein set-point are not influenced by mating status or reproduction.**(A)** Mating status does not affect the 5-HT2A-dependent reduction in yeast consumption (*n* = 9–10 where each replicate is comprised of 10 flies, Two-way ANOVA Genotype: *p* < 0.001 Mating Status: *p* = 0.64 Interaction *p* = 0.14), and **(B)** loss of 5-HT2A does not alter total reproduction, measured as eggs laid over seven days (*n* = 9 and 10 where each replicate is comprised of 5 flies, two-sided *t*-test *p* = 0.23). All experiments were conducted in females. **(C)** *5-HT2A* ^*−/−*^ mutants are long-lived relative to w-; CS controls on a 10% sucrose-only diet (*n* = 188 and 194, log-rank analysis *p* = 0.003).

SUPPLEMENTARY FIGURE S3 Neuronal activation requires red-light stimulation and chronic activation or inhibition of *5-HT2A ^+^* neurons shortens lifespan. Flies expressing CsChrimson in *5-HT2A ^+^* neurons do not show differences in interactions with a **(A)** protein solution (inset: quantification of total interactions, *n* = 23 and 25, two-sided *t*-test *p* = 0.12) or **(B)** sucrose-only solution in the absence of red light activation (inset: quantification of total interactions, *n* = 12 and 18, two-sided *t*-test *p* = 0.09). **(C)** Activation (*n* = 78 and 79, log-rank analysis *p* = 0.01), and **(D)** inhibition of *5-HT2A ^+^* neurons shorten lifespan relative to controls maintained in constant darkness (*n* = 188 and 194, log-rank analysis *p* < 0.001). All experiments were conducted in females.
