# Supplementary figures and images for: The serotonin receptor 5-HT2A modulates lifespan and protein feeding in Drosophila melanogaster
Source: Front Aging. 2022 Dec 1;3:1068455. doi: 10.3389/fragi.2022.1068455 (PMC9751412; doi:10.3389/fragi.2022.1068455)

# Fig. 2 Supplement

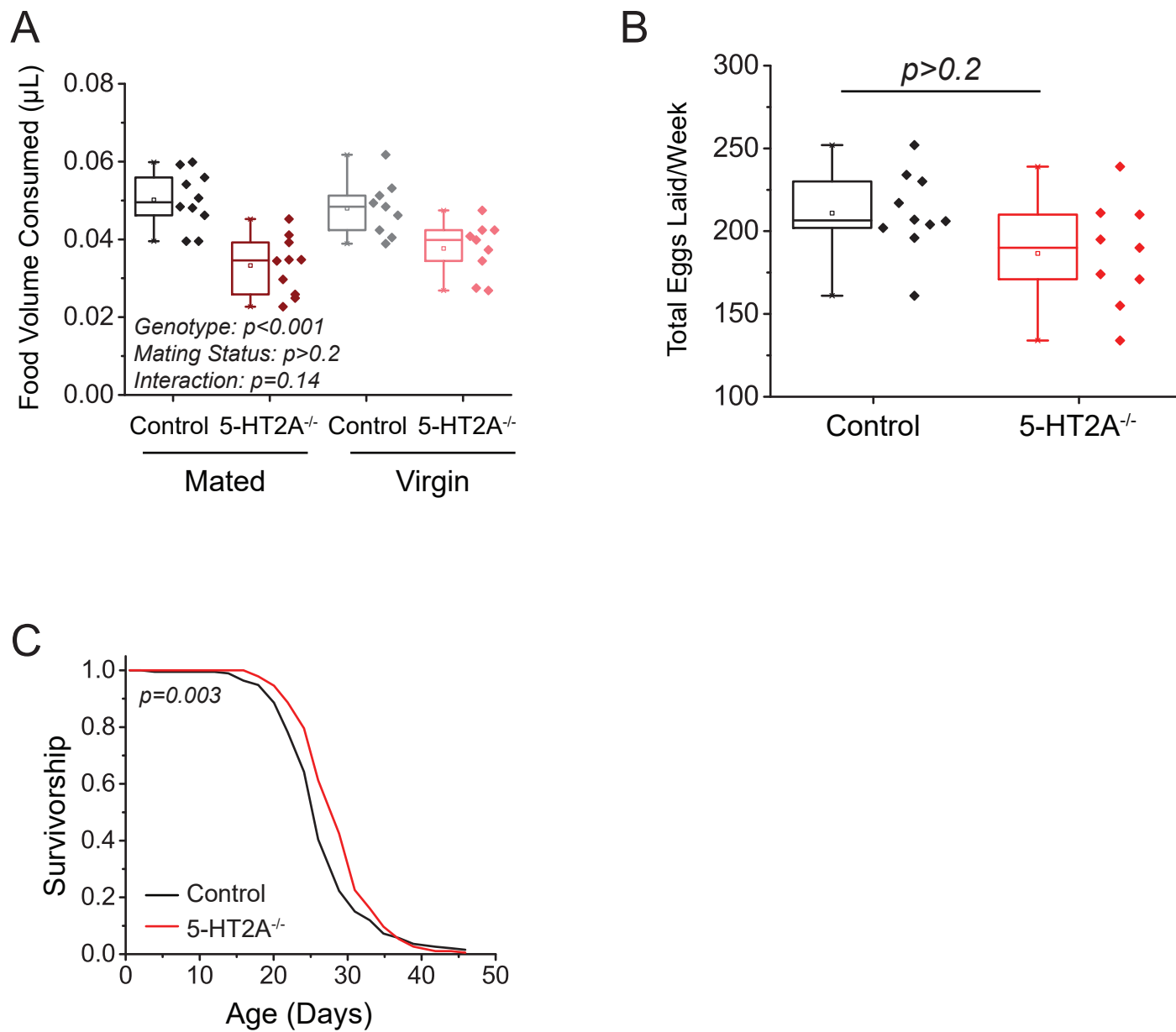

Supplement: Supplementary file 1 [file Image2.pdf]

# Fig. 3 Supplement

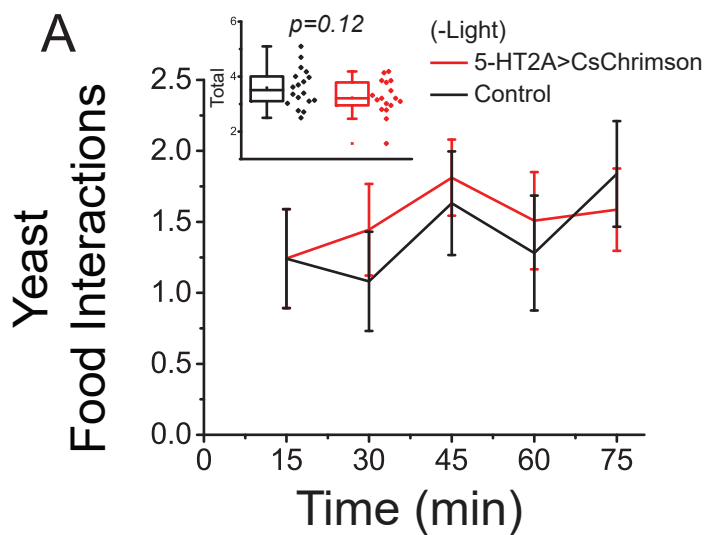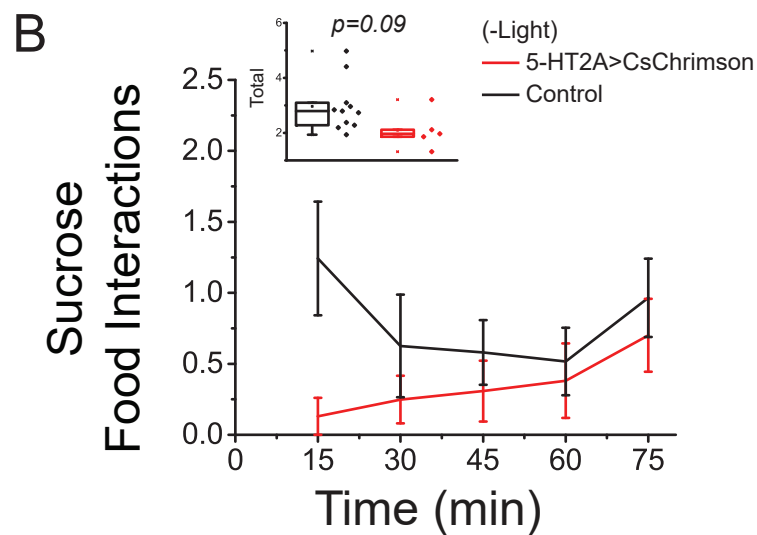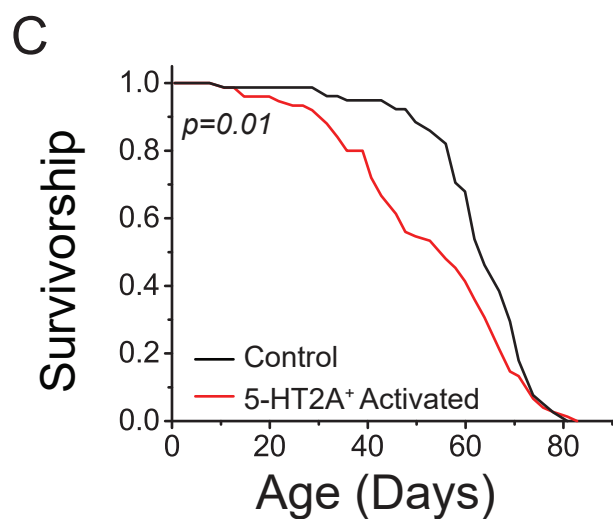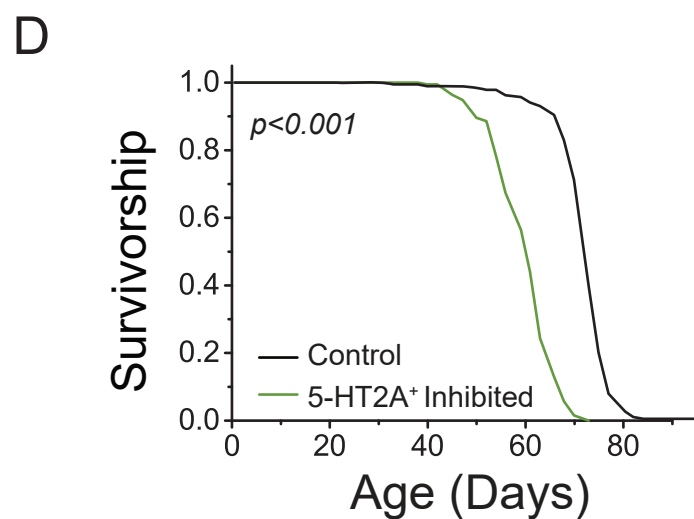

Supplement: Supplementary file 2 [file Image3.pdf]

# Fig. 1 Supplement

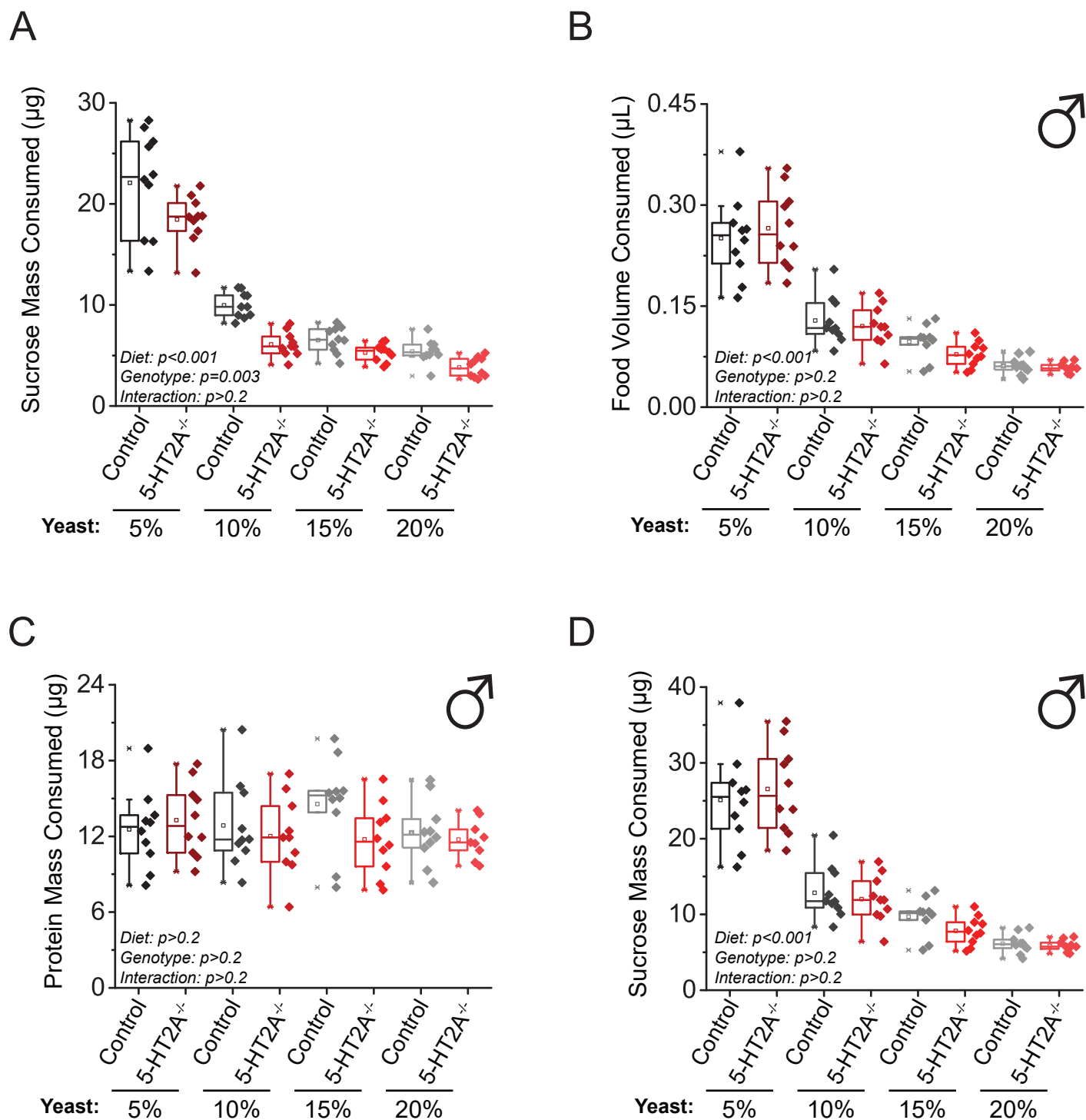

Supplement: Supplementary file 3 [file Image1.pdf]
